# NMDAR-dependent somatic potentiation of synaptic inputs is correlated with β amyloid-mediated neuronal hyperactivity

**DOI:** 10.1186/s40035-021-00260-3

**Published:** 2021-09-08

**Authors:** Yifei Bao, Xin Yang, Yi Fu, Zhengyan Li, Ru Gong, Wei Lu

**Affiliations:** 1 MOE Key Laboratory of Developmental Genes and Human Disease, School of Life Science and Technology, Southeast University, Nanjing, 210096 China; 2 Department of Neurology, Huashan Hospital, Institute for Translational Brain Research, State Key Laboratory of Medical Neurobiology, MOE Frontier Center for Brain Science, Fudan University, Shanghai, 200032 China; 3 Co-Innovation Center of Neuroregeneration, Nantong University, Nantong, 226001 China

**Keywords:** NMDA receptor, Hyperactivity, Somatic modification, Alzheimer’s disease

## Abstract

**Background:**

β Amyloid (Aβ)-mediated neuronal hyperactivity, a key feature of the early stage of Alzheimer’s disease (AD), is recently proposed to be initiated by the suppression of glutamate reuptake. Nevertheless, the underlying mechanism by which the impaired glutamate reuptake causes neuronal hyperactivity remains unclear. Chronic suppression of the glutamate reuptake causes accumulation of ambient glutamate that could diffuse from synaptic sites at the dendrites to the soma to elevate the tonic activation of somatic *N*-methyl-*D*-aspartate receptors (NMDARs). However, less attention has been paid to the potential role of tonic activity change in extrasynaptic glutamate receptors (GluRs) located at the neuronal soma on generation of neuronal hyperactivity.

**Methods:**

Whole-cell patch-clamp recordings were performed on CA1 pyramidal neurons in acute hippocampal slices exposed to TFB-threo-β-benzyloxyaspartic acid (TBOA) or human Aβ_1–42_ peptide oligomer. A series of dendritic patch-clamp recordings were made at different distances from the soma to identify the location of the changes in synaptic inputs. Moreover, single-channel recording in the cell-attached mode was performed to investigate the activity changes of single NMDARs at the soma.

**Results:**

Blocking glutamate uptake with either TBOA or the human Aβ_1–42_ peptide oligomer elicited potentiation of synaptic inputs in CA1 hippocampal neurons. Strikingly, this potentiation  specifically occurred at the soma, depending on the activation of somatic GluN2B-containing NMDARs (GluN2B-NMDARs) and accompanied by a substantial and persistent increment in the open probability of somatic NMDARs. Blocking the activity of GluN2B-NMDARs at the soma completely reversed both the TBOA-induced or the Aβ_1–42_-induced somatic potentiation and neuronal hyperactivity.

**Conclusions:**

The somatic potentiation of synaptic inputs may represent a novel amplification mechanism that elevates cell excitability and thus contributes to neuronal hyperactivity initiated by impaired glutamate reuptake in AD.

**Supplementary Information:**

The online version contains supplementary material available at 10.1186/s40035-021-00260-3.

## Background

Neuronal hyperactivity is a major pathology commonly present in a number of neurological disorders, including Alzheimer’s disease (AD) [1–3]. This featured electrophysiological change in neurons has been implicated in the development of several core symptoms of these diseases [4]. Notably, the aberrant neuronal activity in various neuropsychiatric states is often accompanied by deficits in glutamate transporters (GLTs) in glial cells, which result in the accumulation of ambient glutamate in the extracellular milieu [5], leading to the activation of additional glutamate receptors (GluRs) and thus potentiating the tonic activation of GluRs on surrounding neurons. This enhancement of activation of GluRs by ambient glutamate has been proposed to lead to neuronal hyperactivity [6]. Consistently, Aβ has been reported to induce metabolic disorder by jeopardizing glial cells and in turn lead to neuronal hyperactivity [7]. TFB-threo-β-benzyloxyaspartic acid (TBOA) is an unspecific glutamate uptake blocker that can elicit such accumulation of ambient glutamate and neuronal hyperactivity. Moreover, a recent study has demonstrated that local application of Aβ peptide to hippocampal CA1 neurons can mimic the effect of TBOA in inducing neuronal hyperactivity through initiating the suppression of glutamate reuptake [8]. Despite these findings, how deficits in GLT reuptake in glial cells contribute to neuronal hyperactivity remains largely unknown.

Glutamate is one of the most important neurotransmitters in the central nervous system. Ambient glutamate is implicated in several important neurological processes. Most previous studies have focused on how blockade of glutamate uptake affects synaptic events, including basal neurotransmission and synaptic plasticity [9, 10]. For example, the unspecific glutamate uptake blocker TBOA has been found to prolong the decay time of *N*-methyl-*D*-aspartate receptor (NMDAR)-mediated synaptic currents [11] *via* activation of perisynaptic NMDARs by elevated ambient glutamate. However, the effects of TBOA might be more widespread as extrasynaptic GluRs can also be activated by ambient glutamate [12]. A typical extrasynaptic domain for such receptors is the soma, where in general only a few inhibitory synapses innervate [13]. It is possible that following chronic suppression of the GLTs, the accumulated ambient glutamate could diffuse from synaptic sites at the dendrites to the soma to elevate the tonic activation of somatic NMDARs. However, less attention has been paid to the potential role of tonic activity change of extrasynaptic GluRs, especially GluRs at the subcellular domain proximal to the neuronal soma, in the generation of neuronal hyperactivity.

The elevated level of ambient extracellular glutamate has been shown to both constitutively activate and desensitize GluRs, especially GluRs at the extrasynaptic domains [14–16]. The exact sensitivity of GluR activation and desensitization depends on the GluR subtype [17]. Compared to the activation of AMPA and kainate receptors, the NMDAR activation is much more sensitive to changes in tonic glutamate level. NMDARs are composed of the obligatory GluN1 subunit and regulatory GluN2A-D or GluN3A-B subunits [18, 19]. Among these NMDAR subunits, the GluN2A and GluN2B subunits are essential to the properties of NMDAR. They have a relatively longer intracellular C-tail than the NR1 subunit, which enables them to interact with neighboring postsynaptic density proteins (PSD) [20]. Moreover, the GluN2A- and GluN2B-containing NMDARs are preferentially located at the synaptic and extrasynaptic sites, respectively [21]. Notably, the GluN2B-containing NMDARs (GluN2B-NMDARs) possess relatively higher sensitivities to glutamate and lower open probability than GluN2A-containing NMDARs (GluN2A-NMDARs) [22]. The different subtype distributions may therefore allow neurons to better integrate extrasynaptic inputs and respond to changes in the extracellular milieu. In acute hippocampal slices, for example, NMDARs in hippocampal CA1 pyramidal cells can be persistently activated by ambient extracellular glutamate [23, 24]. The tonic activation of NMDARs is also thought to primarily affect the cell excitability. For instance, the increase of the magnitude of tonic NMDAR currents which occurs with depolarization, due to the relief of voltage-gated Mg^2+^ blockade of NMDAR channels, can increase the tendency of the cell toward regenerative depolarization [25]. Previous findings have also suggested that the tonic NMDAR activity at the soma plays an important role in somatic generation of action potentials (APs). For example, bath application of a NMDAR blocker *D*,*L*-APV reduces the population spike recorded at the pyramidal cell layer where cell bodies of the pyramidal neurons are located, while having no effect on the field excitatory post-synaptic potentials (EPSPs) recorded in the dendritic region [24]. Since the population spike is a measure of the number of pyramidal cell discharges, this study suggests that the tonic activation of somatic NMDARs facilitates neuronal AP discharge at the soma.

As the neuronal soma receives very few excitatory glutamatergic inputs, NMDARs located at the soma are largely extrasynaptic GluN2B-NMDARs. Recently, we have revealed a novel plasticity rule that specifically occurs at the soma [30]. In this study, we set out to determine whether the elevated tonic activation of GluN2B-NMDARs at the soma, elicited by suppression of glutamate reuptake, employs a similar somatic potentiation mechanism to amplify dendritic inputs and in turn contributes to the induction of Aβ-mediated neuronal hyperactivity, which is believed to be associated with the circuit dysfunction that characterizes the early stages of AD.

## Materials and methods

### Acute hippocampal slice preparation

Male Sprague–Dawley rats of 2–3 weeks old were deeply anesthetized with ethyl ether and decapitated. Brains were removed immediately and placed in ice-cold artificial cerebrospinal fluid (ACSF) containing (in mM) 126 NaCl, 2.5 KCl, 1CaCl_2_, 1 MgCl_2_, 1.25 KH_2_PO_4_, 26 NaHCO_3_, and 20 glucose, pH 7.2–7.4. Hippocampal coronal slices of 350-μm thick were obtained using a Leica VT1000 S vibrating blade microtome, transferred to warm (34 °C) ACSF and recovered for at least 1 h. ACSF solutions were bubbled with 95% O_2_/5% CO_2_ throughout the experiment. All experiments were performed in accordance with guidelines of Animal Care and Committee of Southeast University.

### Hippocampal slice recording

EPSPs were recorded from acute brain slices in a current-clamp mode, with whole-cell recording using borosilicate glass pipettes containing (in mM) 135 K-gluconate, 5 KCl, 2 NaCl, 0.2 EGTA, 10 HEPES, 5 ATP, with pH adjusted to 7.2 by KOH. Bicuculline methiodide (BMI, 10 μM; Tocris, Bristol, England) was routinely added to the bath solution to block the type A gamma-aminobutyric acid (GABA_A_) receptor-mediated inhibitory synaptic currents. Neurons in the CA1 stratum radiatum were identified under infrared differential interference contrast (IR-DIC) video microscopy using an upright microscope (BX51WI, Olympus Corporation, Tokyo, Japan) and recorded with an Axopatch-700B amplifier (Molecular Devices, San Jose, CA). Data were sampled at 10 kHz and low-pass filtered at 2 kHz. The pipette resistances in the bath for somatic recording ranged 4–6 MΩ, and for dendritic patch recording ranged 12–15 MΩ. The series access resistance ranged 8–30 MΩ and was monitored during recording for fear of resealing of the ruptured membrane. Cells with changes in series access resistance > 30 MΩ at any time during recordings were removed from the analysis. The amplitude of basal evoked EPSPs (eEPSPs) was set between 2 and 5 mV by adjusting the current intensity of test stimuli. For all plasticity experiments, the EPSP amplitude under the baseline condition was compared with that during the last 10 min of recording. Detailed  data regarding the absolute values of membrane voltage, membrane resistance, and number of animals are provided for each experiment. For single-channel recordings, 50 μM NMDA and 35 μM glycine were used to elicit activities of NMDARs. The pipettes were filled with Mg^2+^-free ACSF, and patch-clamp was performed in a cell-attached mode. In order to puff NMDAR antagonist locally onto the soma or dendrite, Picospritzer (Intracell Co., England) was employed to control the speed, duration and pressure of puffings. Data were captured with the Clampex 10.4 software and analyzed using the Clampfit 10.4 software (Molecular Devices, San Jose, CA).

### Aβ oligomerization and treatment solutions

Aβ_1–42_ oligomers were prepared as described previously [26] and identified by Western blotting from a standard 15% SDS-PAGE, using an antibody against Aβ_1–42_ (Cell Signaling Technology, Danvers, MA). The Aβ_42–1_ peptide was prepared identically. In electrophysiology experiments, Aβ_1–42_ aliquots were diluted into extracellular solutions to a final concentration of 500 nM.

### Western blotting

Five microliters of either Aβ_1–42_ oligomers or Aβ_42–1_ peptide (80 μM) were mixed with 1 × loading buffer and separated by 15% SDS-PAGE without boiling. Then they were transferred to nitrocellulose membranes. The membranes were blocked for 1 h in a solution of 3% (*w*/*v*) BSA diluted in TBS-T buffer (20 mM Tris/HCl [pH 7.4], 150 mM NaCl and 0.1% Tween-20), and incubated overnight with rabbit monoclonal antibody for Aβ_1–42_ (D9A3A) (Cell Signaling Tecnnology; 1:1000), which was produced by immunizing animals with a synthetic peptide corresponding to residues near the carboxy terminus of human β-amyloid (1–42) peptide. HRP-conjugated goat anti-rabbit IgG (SunShine Bio, Nanjing, Jiangsu, China) was used as the secondary antibody (1:500). Finally, the signals were developed using the Super Signal West Pico Chemiluminescent Substrate kit (Thermo Fisher Scientific, Waltham, MA). Molecular mass was assessed by the Rainbow molecular weight markers (Bio-Rad, Hercules, CA).

### Statistical analysis

Data were analyzed with Student’s *t* test or Kolmogorov–Smirnov tests using the SPSS software (IBM, Armonk, NY). Differences were regarded as significant when *P* < 0.05.

## Results

### TBOA induces neuronal hyperactivity

**Fig. 1:**
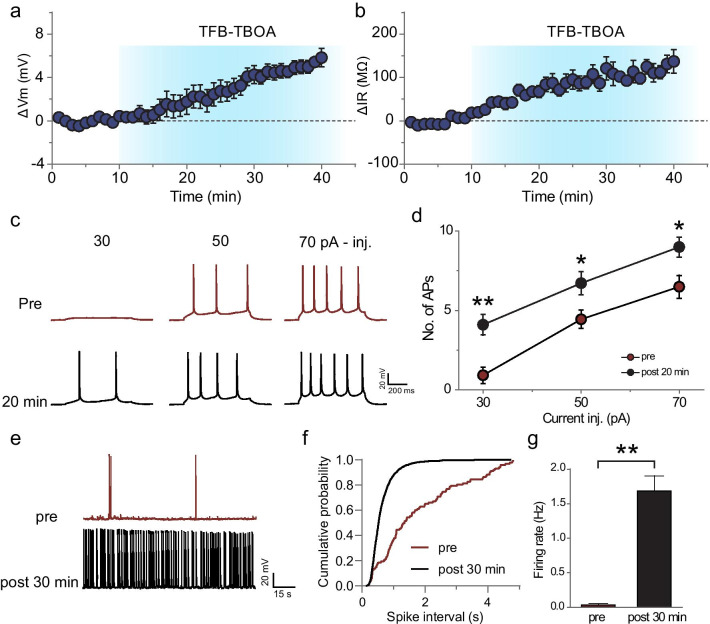
TBOA changes the membrane property and induces neuronal hyperactivity. **a**, **b** The time course and magnitude of changes in RMP (**a**) and input resistance (**b**) after TFB-TBOA (100 nM) treatment. Note that the input resistance was measured by a single − 20 pA injection. The blue-shaded area indicates the time period of TFB-TBOA treatment. **c** Examples of AP firing triggered by a 600-ms current injection of either 30, 50 or 70 pA before (top) and 20 min after (bottom) TFB-TBOA (100 nM) treatment. **d** Summary data showing significant increase in the number of APs elicited by somatic current injection 20 min after TFB-TBOA (100 nM) treatment. **e** Example traces showing spontaneous firing of recorded CA1 pyramidal neurons before (RMP − 66.94 mV) and after (RMP was − 59.17 mV) TFB-TBOA treatment. AP number was dramatically increased. (**f**) Cumulative distribution of inter-spike intervals (ISIs). **g** Statistical histogram showing significant enhancement of firing rate after TFB-TBOA treatment. Data are means ± SEM; *t* test for pre- versus post-treatment comparison; *P < 0.05; **P < 0.01

As TBOA has been reported to mimic Aβ in the effect on both neuronal excitability and activity-dependent synaptic plasticity in vitro [8, 27, 28], we start with examining the effect of TBOA on the neuronal intrinsic excitability. Acute hippocampal slices were obtained from rats of postnatal 2–3 weeks of age. The whole-cell patch-clamp recordings were performed to detect membrane properties and cell excitability in CA1 pyramidal neurons. Persistent perfusion of TFB-TBOA (100 nM) led to progressive depolarization from resting membrane potential (RMP) in hippocampal CA1 neurons (Δ*V*m, 4.84 ± 0.16 mV, *n* = 10 slices from 7 rats, *P* < 0.01; Fig. 1a). The averaged RMP at baseline stage was − 63.53 ± 1.02 mV, and 30 min after TFB-TBOA treatment the membrane voltage was − 58.69 ± 0.33 mV. Compared to the RMP at the baseline stage, the hippocampal CA1 neurons attained 5 mV more depolarization after 30 min of TFB-TBOA (100 nM) treatment (Fig. 1a). This alteration in RMP was accompanied by an increase in the input resistance (*R*_in_) in the same recorded cells (Δ*R*_in_, 113.13 ± 4.50 MΩ, *n* = 10 slices from 7 rats, *P* < 0.01; Fig. 1b and Additional file 1: Fig. S1). The averaged *R*_in_ at baseline stage was 380.23 ± 7.02 MΩ and that after 30 min of TFB-TBOA treatment was 493.35 ± 10.79 MΩ. These results indicate that blockade of glutamate reuptake induces hippocampal CA1 neurons toward more depolarization.

To confirm the elevated level of excitation in recorded cells following TFB-TBOA treatment, we next determined both the threshold of AP generation and the firing rate in hippocampal CA1 neurons. We examined the possible change in AP threshold by injecting an epoch of current (600 ms) with different intensities (30, 50, 70 pA) in the recorded cells (Fig. 1c). Compared to controls, the TBOA-treated CA1 pyramidal cells displayed more firing spikes at the injection of the same intensity of current (Fig. 1d). In a separate set of experiments, we further examined the firing rate in hippocampal CA1 neurons. Representative traces in Fig. 1e shows that the AP firing rate is very low under the baseline condition. However, GLT-1 blockade with TFB-TBOA (100 nM) substantially enhanced the AP firing rate from 0.03 ± 0.02 to 1.68 ± 0.22 Hz, which indicates significant potentiation in the firing rate of recorded cells (*n* = 8 from 5 rats, *P* < 0.01; Fig. 1g). Accordingly, cumulative probability distributions of spike intervals revealed a dramatic left-shift, suggesting a significant shortening of spike intervals (*P* < 0.01; Fig. 1f). In conclusion, our results indicate that the GLT defunctionalization causes neuronal membrane depolarization and hyperactivity in hippocampal CA1 pyramidal cells, which is consistent with a previous finding [29].

### Neuronal hyperactivity depends on somatic NMDAR activity

**Fig. 2:**
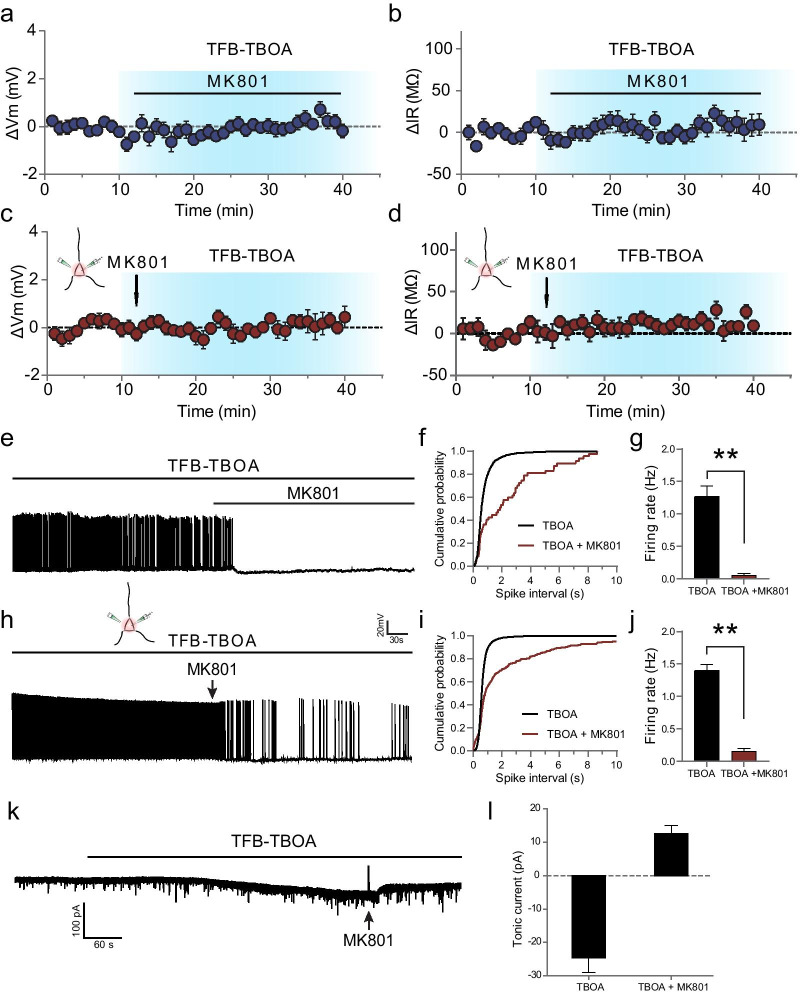
The TBOA-induced changes in neuronal hyperactivity depend on the activation of NMDARs at the soma. **a–d** Time course and magnitude of the change in RMP (**a**, **c**) and input resistance (**b**, **d**). TFB-TBOA (100 nM) was applied *via* perfusing the slices (**a**, **b**) or *via* pressure puffing locally on the soma of recorded neurons (**c**, **d**). No significant changes in input resistance and RMP were observed after co-application of MK-801 (50 μM) and TFB-TBOA (100 nM). The blue-shaded area indicates the time period of TFB-TBOA treatment. Horizontal bars indicate drug application. The insets illustrate the local application of MK-801 *via* a pipette placed close to the soma of the recorded neuron. The arrow indicates the timepoint of somatic MK-801 application. **e** Example traces showing spontaneous firing of recorded CA1 pyramidal neurons before and after co-application of MK-801 and TFB-TBOA. Whole slice perfusion with MK-801 totally abolished the spontaneous firing. **f** Cumulative distribution of ISIs. **g** MK-801, when co-applied with TFB-TBOA, reversed the TBOA-induced potentiation in firing rate. **h**–**j** Same as in (**e–g**) except that MK-801 was locally applied on the soma. The duration of local MK-801 application was 500 ms. **k** Representative samples showing that blockade of GLTs (TFB-TBOA, 100 nM) induced an inward shift in the holding current and local application of MK-801(50 μM) to the soma partially reversed the holding current. **l** Summary data of the shift of mean holding current. Data are means ± SEM. *t* test, **P < 0.01

NMDAR hyperactivation is a characteristic feature in AD [28]. We next examined the possible role of NMDARs in the TBOA-induced neuronal hyperactivity. For this purpose, we repeated the above experiments on neuronal membrane properties under the condition that NMDAR activity was blocked by the NMDAR channel blocker MK-801 (50 µM). We found that application of MK-801 through whole-slice perfusion reversed the alterations of RMP and *R*_in_ (RMP at baseline stage was − 69.13 ± 0.38 mV, and after 30 min of MK-801 treatment the membrane voltage was − 68.92 ± 0.53 mV; *R*_in_ at baseline stage was 489.69 ± 13.83 MΩ and that after 30 min of MK-801 treatment was 487.86 ± 17.25 MΩ; *n* = 9 from 7 rats; Fig. 2a, b), suggesting a crucial role of NMDARs in the TBOA-induced changes in membrane property.

It has been reported that under basal conditions, blockade of NMDARs at the soma rather than at the dendritic tree (where activated synapses locate) compromises the population spike generation [24]. As the population spike is a measure of the number of discharging pyramidal cells, this finding points to the notion that tonic activation of NMDARs at the soma plays an important role in somatic AP generation. To test whether tonic activation of NMDARs at the soma is responsible for the neuronal hyperactivity shown above, MK-801 (50 µM) was locally applied for 500 ms *via* pressure puffing to the soma of the recorded CA1 neurons (Fig. 2c, d). Notably, the TBOA-induced changes in RMP and *R*_in_ were absent following MK-801 treatment, indicating total reversal of neuronal hyperactivity (RMP at baseline stage was − 65.53 ± 0.15 mV and 30 min after brief MK-801 treatment the membrane voltage was − 65.37 ± 0.13 mV; *R*_in_ at baseline stage was 469.13 ± 15.68 MΩ and 30 min after brief MK-801 treatment *R*_in_ was 479.65 ± 15.71 MΩ, *n* = 10 from 7 rats). Collectively, these results highly suggest that the neuronal hyperactivity induced by GLT blockade depends on the activity of NMDAR at the soma.

In a separate set of experiments, we also investigated the possible changes in neuronal hyperactivity by examining the firing rate in hippocampal CA1 neurons. Consistently, we detected substantially enhancement in the AP firing rate following GLT-1 blockade with TFB-TBOA (100 nM), indicating that the firing rate of recorded cells was significantly potentiated (1.4 ± 0.09 Hz, *n* = 5 from 5 rats, *P* < 0.01; Fig. 2e–j). The TBOA-induced potentiation in AP firing rate was, however, largely abolished by MK-801 (50 µM) either *via* whole-slice perfusion (0.05 ± 0.03 Hz, *P* < 0.01; Fig. 2e, g) or *via* local application on the soma (0.15 ± 0.09 Hz, *n* = 6 from 5 rats, *P* < 0.01; Fig. 2h, j). Accordingly, the cumulative probability distributions of spike intervals revealed a dramatic right-shift, suggesting a significant prolongation of spike intervals (*P* < 0.01, Fig. 2f, i).

Our results demonstrate the crucial role of NMDARs at the soma in TBOA-induced potentiation of AP firings. Next, we examined whether TBOA can also induce modification of the tonic current mediated by NMDARs located at the soma. For this purpose, we performed whole-cell recording in CA1 neurons while voltage-clamping these cells at − 45 mV. The bath-applied TFB-TBOA (100 nM) induced an inward shift in *I*_holding_ (− 24.60 ± 4.38 pA, *n* = 11 from 7 rats; Fig. 2k, l), which could be partially reversed by local application of NMDAR antagonist MK-801 to the soma of recorded neurons (12.61 ± 2.34 pA), suggesting that activation of somatic NMDARs contribute to the TBOA-induced enhancement in tonic currents.

### Somatic modification of dendritic inputs

**Fig. 3:**
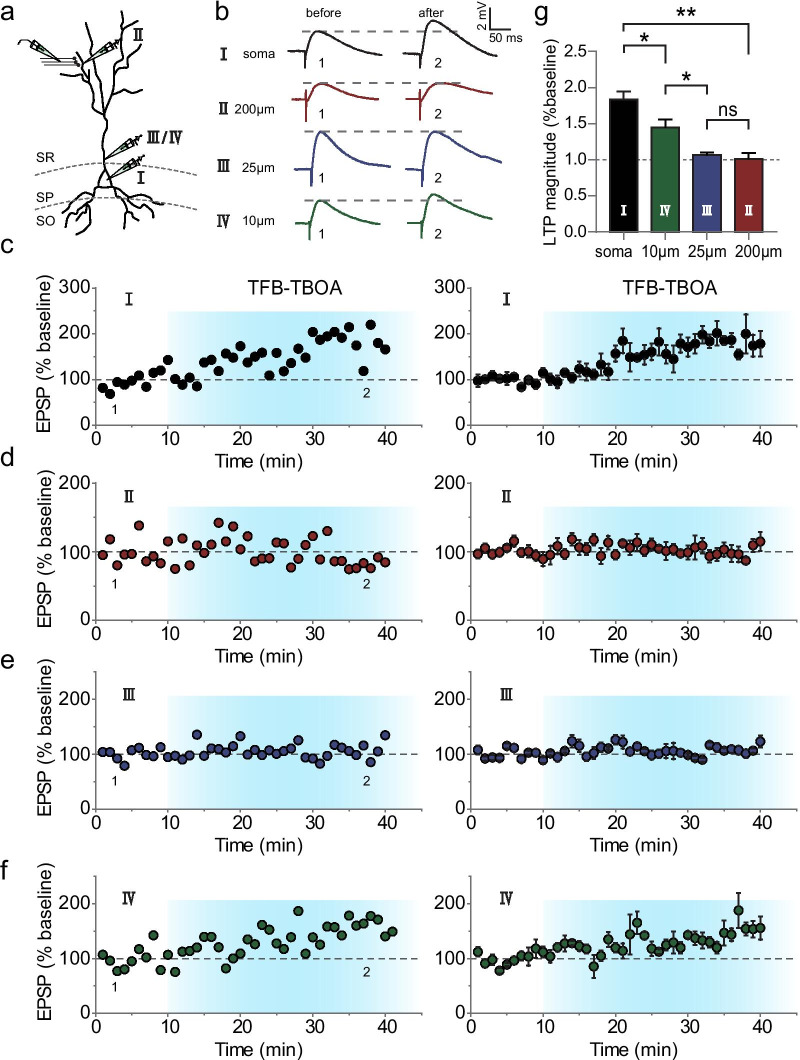
TBOA elicits somatic amplification of dendritic inputs. **a** Illustration of stimulating and recording paradigm. Patch recordings were performed at the soma (I) or at the dendrites with different distances from the soma (II, 200 μm; III, 25 μm; IV, 10 μm). Low-frequency (0.1 Hz) basal stimulation was delivered at site II. **b** Sample traces showing the averaged EPSPs recorded at the soma or at the dendrites with different distances from soma. Sample EPSP traces are the average of 30 consecutive events recorded at the time marked by the numbers (1 or 2) from (**c**–**f**). **c**–**f** Graphs showing the time course and magnitude of the change in normalized EPSPs from a sample recording at the soma marked by I (**c**) or at dendrites located at different distances (marked by II, III, IV) from the soma (**d**–**f**). The TFB-TBOA (100 nM)-elicited potentiation in normalized EPSPs was only observed at the soma or at the dendrite located at site IV (10 μm distance) and was absent at site II (200 μm distance) or site III (25 μm). Notably, the potentiation magnitude in recordings at site IV (10 μm distance) was lower than that recorded at the soma. Left column, data from a sample recording. Right column, summary of results from all experiments. **g** Statistical histogram showing attenuation of TBOA-potentiated somatic EPSPs along the apical dendrite. Somatic EPSP potentiation was attenuated to the baseline level at apical dendrites within 25 μm from the soma. Data are means ± SEM. *t* test, *P < 0.05; **P < 0.01, ns, no significance

Our recent study reveals a unique form of plasticity rule that potentiates dendritic inputs selectively at the soma [30]. In particular, this somatic modification depends on the NMDAR activity at the soma. We thereby examined if GLT blockade could trigger the somatic NMDAR-dependent modification of dendritic inputs, which may in turn contribute to the neuronal hyperactivity observed following TFB-TBOA treatment. Hippocampal slices were perfused with the specific antagonist of GABA_A_ receptor BMI (10 μM) to isolate evoked currents mediated by glutamate receptors. The eEPSPs were elicited by bipolar stimulating electrodes (made from the borosilicate theta glass) placed at a distance of 200 μm from the soma (Fig. 3a). The stimulating intensity was adjusted to produce eEPSPs at 2–5 mV. We found that the eEPSPs underwent a progressive and substantial potentiation following TBOA (100 nM) application (normalized potentiation magnitude, 1.84 ± 0.04, *n* = 7 from 7 rats, *P* < 0.01; Fig. 3b, c).

To test whether the potentiation also occurs at the dendrite where the activated synapses are usually located, we performed serial patch-clamp recordings at the apical dendrite at different distances from the soma (Fig. 3d–f). We found that TFB-TBOA (100 nM) treatment did not produce any change in dendritic eEPSPs located at 200 μm (site II, normalized potentiation magnitude, 1.01 ± 0.03, *n* = 4 from 4 rats, *P* = 0.76; Fig. 3d) or 25 μm (site III, normalized potentiation magnitude, 1.07 ± 0.03, *n* = 4 from 4 rats, *P* = 0.15; Fig. 3e) from the soma, indicating the absence of EPSP potentiation even at the site closer to the soma. To identify the precise location of the eEPSPs potentiation, we further performed patch-clamp recordings at the apical dendrite at 10 μm distance from the soma (site IV; Fig. 3f). Notably, our recordings revealed a significant potentiation at this dendritic site (normalized potentiation magnitude, 1.46 ± 0.06, *n* = 4 from 4 rats, *P* < 0.01), which was however lower than that recorded at the soma (*P* < 0.05). Collectively, these results support the notion that the potentiation of synaptic inputs originates at the soma and undergoes progressive attenuation along the apical dendritic shaft away from the soma.

The  small-conductance Ca^2+^-activated K^+^ channels (usually known as SK channels) have been reported to couple to NMDARs both at the synapse and at the axonal dendrite [31, 32]. Therefore, it is possible that the TBOA-induced increase of permeability to Ca^2+^ through NMDARs activates SK channels and thus regulates hyperactivity. To test this, we performed a set of new experiments of serial patch in the presence of an SK channel blocker apamin (100 nM). A serial of patch recordings on the soma or on the dendrites at 10 μm and 200 μm distance from the soma revealed absence of effect of apamin on the TBOA-induced somatic amplification of dendritic inputs (Additional file 1: Fig. S2), suggesting that the SK channel is not involved in this process.

### Somatic modification depends on the GluN2B-NMDARs at the soma

**Fig. 4:**
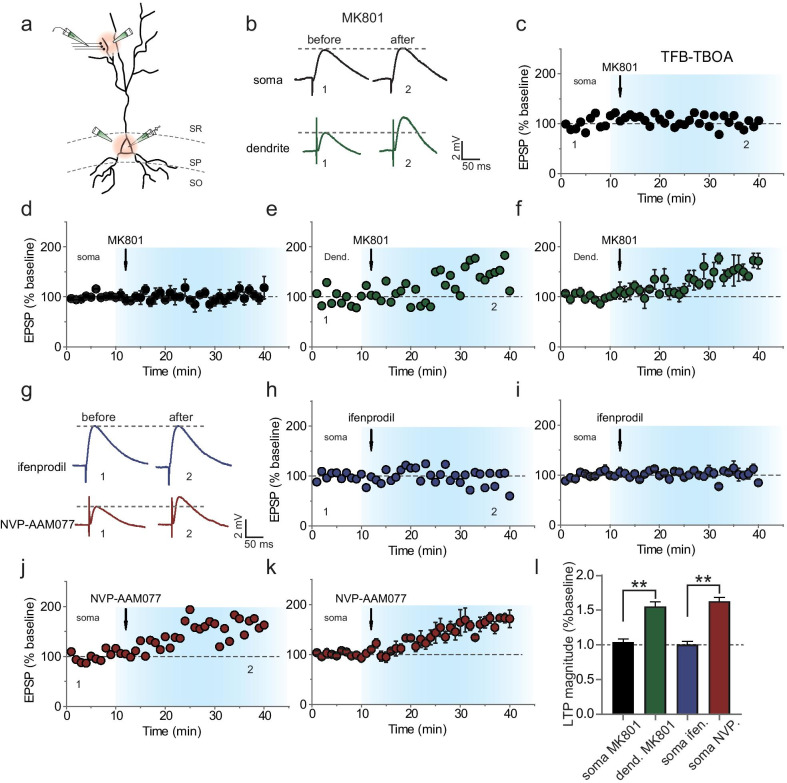
Somatic amplification of dendritic inputs depends on the activity of GluN2B-NMDARs located at the soma. **a** Illustration of stimulating and recording paradigm. Note that the MK-801 was either locally applied to the soma or to the dendrite of the recorded neuron where the stimulating electrode was placed (200 μm distance from the soma). **b** Sample traces showing averaged EPSPs recorded at the soma or at dendrites in hippocampal CA1 pyramidal neurons. Sample EPSP traces are the average of 30 consecutive events recorded at the time marked by the numbers (1 or 2) from **c-f**. MK-801 (50 μM) was locally applied with TFB-TBOA (100 nM) either at the soma or at the dendrite. **c–f** MK-801 applied at the soma totally abolished the TBOA-elicited EPSP potentiation. **c**, **e** Results from a single experiment displaying the time course and magnitude of the normalized EPSPs. **d**, **f** Summary of results from all experiments as that in (**c**) and (**e**), respectively. MK-801 applied to the dendrite failed to affect the TBOA-induced potentiation in normalized EPSPs, while MK-801 applied to the soma reversed the EPSP potentiation induced by TBOA. **g** Sample traces showing the effect of suppressing GluN2B- or Glu2NA-NMDARs on the average EPSPs recorded at the soma of hippocampal CA1 pyramidal neurons treated with TFB-TBOA (100 nM). Note that the antagonist of GluN2-NMDARs was locally applied to the soma. **h–k** Antagonist of GluN2B-NMDARs, but not of GluN2A-NMDARs applied at the soma, totally abolished the TBOA-elicited EPSP potentiation. Results from a single experiment (**h**, **j**) and summarized results (**i**, **k**) displaying the time course and the magnitude of the normalized EPSPs. **l** Statistical histogram showing the effect of locally applied NMDAR antagonists. MK-801 (50 μM) or ifenprodil (3 μM) applied to the soma totally reversed the TBOA-induced EPSP potentiation. Data are means ± SEM, *t* test, **P < 0.01

As the activity of NMDARs at the soma has recently been reported to be important for the somatic modification of dendritic inputs [30], we next tested whether the somatic modification caused by GLT blockade also depends on the activity of somatic NMDAR. Two minutes after TFB-TBOA treatment, MK-801 (50 μM) was applied to the soma with pressure injection for 500 ms to block the activity of somatic NMDARs (Fig. 4a). We found that this brief blockade of somatic NMDARs totally abolished the potentiation of eEPSPs elicited by TFB-TBOA treatment (normalized potentiation magnitude, 1.04 ± 0.03, *n* = 7 from 7 rats, *P* = 0.28; Fig. 4b–d). In a separate set of experiments, we additionally applied MK-801 (50 μM) *via* pressure injection to the dendrite that is proximal to the site of electrical stimulation. We found that this brief blockade of NMDAR activity at the stimulated dendrite failed to exert any significant influence on the potentiation of the dendritic input (normalized potentiation magnitude, 1.55 ± 0.04, *n* = 5 from 5 rats, *P* < 0.01; data on TBOA-induced EPSPs in Fig. 3c were used for comparison; Fig. 4b, e and f). Notably, the concentration of MK-801 used here was much higher than that usually used (1–10 μM). To verify whether MK-801 at 10 μM could exert similar blocking effect as that by 50 μM MK-801 (Additional file 1: Fig. S3), we repeated the experiments using 10 μM MK-801 to locally block the activity of NMDARs located at the soma. We found that under this condition the change in TFB-TBOA-induced potentiation in EPSPs could still be reversed in the recorded neurons (normalized potentiation magnitude, 1.06 ± 0.05, *n* = 5 from 4 rats). Another concern on MK-801 at 50 μM was that it might have multiple unspecific targets beyond NMDARs, including various calcium channels. To examine the possible involvement of calcium channels in the TFB-TBOA-induced potentiation of EPSPs, we monitored EPSPs during TFB-TBOA application in the presence of L-type calcium blocker nimodipine (10 μM) and T-type calcium blocker NiCl_2_ (100 μM) (Additional file 1: Fig. S4). No effects of the calcium channel blockers were observed, thus occluding the involvement of these calcium channels in the somatic potentiation of synaptic inputs. In other words, the inhibitory effect of  50 μM MK-801 on the somatic potentiation was not mediated by its action on calcium channels. Collectively, these results indicate that somatic modification induced by GLT blockade requires activation of NMDARs at the soma.

As the soma of hippocampal CA1 neurons receives very few excitatory synaptic inputs, most NMDARs located at the soma are extrasynaptic by definition and are mainly GluN2B-NMDARs [21]. We next tested whether the somatic modification of dendritic inputs selectively depends on the GluN2B-NMDARs at the soma. For this purpose, we pressure-puffed the selective antagonist of GluN2B-NMDARs, ifenprodil (3 μM), on the soma shortly after TFB-TBOA treatment. Again, this brief blockade of GluN2B-NMDARs completely abolished the potentiation of eEPSPs (normalized potentiation magnitude, 1.00 ± 0.04, *n* = 6 from 6 rats, *P* = 0.77; Fig. 4g–i). In contrast, the antagonist of GluN2A-NMDARs, NVP-AAM077 (125 pM), failed to display any significant effect on TBOA-induced potentiation of eEPSPs (normalized potentiation magnitude, 1.63 ± 0.04, *n* = 6 from 6 rats, *P* < 0.01; Fig. 4g, j, k). Taken together, our data support that the somatic potentiation of dendritic inputs depends on the activation of GluN2B-NMDARs at the soma.

### Aβ-mediated neuronal hyperactivity depends on the elevated somatic NMDAR activation

**Fig. 5:**
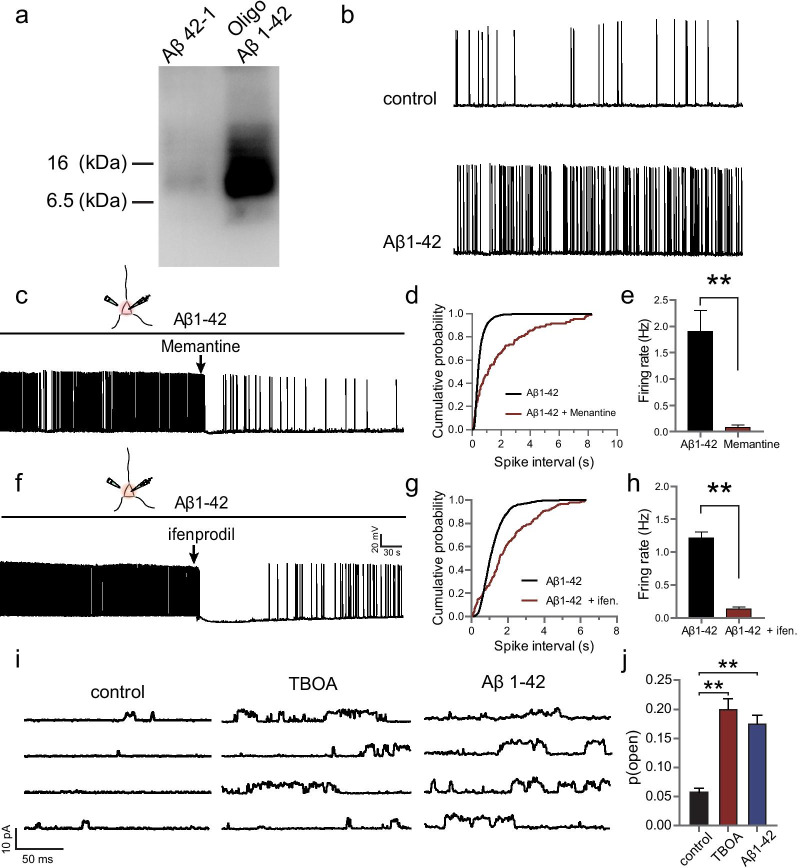
Aβ-mediated neuronal hyperactivity depends on the elevated somatic NMDAR activation. **a** Synthetic human Aβ_1–42_ peptide oligomerization was confirmed by Western blots. Aβ_42–1_ peptide was used as a control. **b** Example traces showing the spontaneous firing of recorded CA1 pyramidal neurons before (RMP =  − 65.97 mV) and after (RMP =  − 60.89 mV) Aβ_1–42_ peptide (500 nM) treatment for 2 h. A dramatic enhancement of firing rate of hippocampal CA1 pyramidal neurons was observed in the Aβ-incubated slices. **c** The increased neuronal firing was reduced to the baseline level by locally applying an open NMDAR antagonist memantine (0.1 mM) to the soma of the recorded neuron. RMP was − 57.53 mV before memantine treatment and − 59.23 mV after memantine treatment. **d** Cumulative distribution of ISIs. **e** Statistical histogram showing that memantine significantly suppressed the Aβ_1–42_–induced enhancement of firing rate. **P < 0.01. **f**–**h** Same as in (**c**–**e**) except that ifenprodil (3 μM) was locally applied to the soma of recorded neurons. RMP was − 56.62 mV before ifenprodil treatment and − 65.24 mV after ifenprodil treatment. **i** Representative traces showing a dramatic increase in single NMDAR opening in the control, TBOA- or Aβ_1–42_-treated hippocampal pyramidal neurons. Cell-attached single-channel recording configuration was employed to record opening of NMDARs located on the soma of the recorded neuron. **j** Statistical histogram showing that both TBOA and Aβ_1–42_ dramatically elevated the open probability of somatic NMDARs. Data are means ± SEM, **P < 0.01, *t* test

Amyloid plaques are the pathological hallmark of AD, and extensive research has identified a causal link of Aβ to AD [33–37]. Numerous studies have also indicated that the impairment of GLTs could be another key feature of AD [38, 39]. These two symptoms are inter-related as the Aβ_1–42_ peptide can slow down the clearance of synaptically released glutamate by inducing mislocalization of astrocytic GLT-1, the major GLT in the adult brain [40], a process which has been employed to model several aspects of AD [41]. Recently, an elegant study has reported that the Aβ-mediated suppression of GLT contributes to the neuronal hyperactivity in an AD model [8]. Here, we revealed that TFB-TBOA (100 nM), an unspecific glutamate uptake blocker, can induce neuronal hyperactivity that depends on somatic NMDAR activation. Collectively, these findings motivated us to evaluate the possible role of somatic NMDAR activation and related somatic modification in Aβ-mediated neuronal hyperactivity. To examine this possibility, we synthetized human Aβ_1–42_ oligomers [26], and confirmed them by Western blots (Fig. 5a). The hippocampal slices were then incubated with Aβ_1–42_ (500 nM) peptide for 2 h. We found that spontaneous firings of the CA1 pyramidal neurons were significantly potentiated following Aβ_1–42_ incubation, indicated by decreased firing interval and dramatically increased firing frequency (1.9 ± 0.4 Hz, *n* = 13 from 6 rats, *P* < 0.01; Fig. 5b). Notably, this neuronal hyperactivity was totally abolished by pressure puffing of memantine (0.1 mM) for 500 ms, an open NMDAR blocker clinically used for AD treatment, to the soma of recorded neurons (0.09 ± 0.04 Hz, *n* = 6 from 6 rats, *P* < 0.01; Fig. 5c–e). Similar observations were detected when ifenprodil (3 μM) was locally applied to the soma of recorded neurons (before: 1.22 ± 0.08 Hz; after: 0.14 ± 0.03 Hz, *n* = 6 from 6 rats, *P* < 0.01; Fig. 5f–h). These data indicate that the Aβ_1–42_-induced neuronal hyperactivity depends on the activation of GluN2B-NMDARs at the soma.

Tonic activation of somatic NMDARs is important for the generation of APs at the soma [24]. GLT dysfunction in glial cells leads to accumulation of glutamate at the extracellular milieu, which may in turn activate the somatic NMDARs. To test this hypothesis, we perfused the hippocampal slices with TBOA (100 nM) or Aβ_1–42_ (500 nM) and performed cell-attached single-channel recording of NMDARs at the soma (Fig. 5i). Strikingly, we found that following the TFB-TBOA or Aβ_1–42_ treatment, the open probability of the clamped somatic NMDAR was dramatically increased (control: 0.06 ± 0.01, *n* = 5 from 5 rats; TBOA: 0.20 ± 0.02, *n* = 5 from 4 rats, *P* < 0.01; Aβ_1–42_: 0.18 ± 0.02, *n* = 5 from 5 rats, *P* < 0.01; Fig. 5j). Notably, this enhancement in NMDAR opening probability persisted either for at least 30 min after TBOA treatment or 2 h after Aβ_1–42_. These findings indicate that GLT blockade improves sustained opening of somatic NMDARs.

### Aβ-mediated somatic potentiation of dendritic inputs

**Fig. 6:**
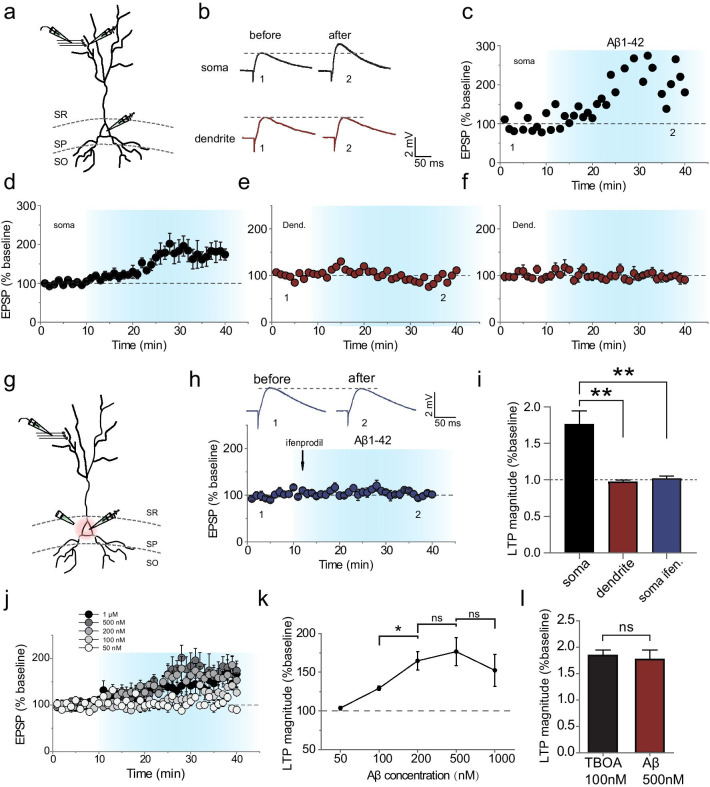
Aβ-mediated somatic potentiation of dendritic inputs. **a** Illustration of the stimulating and recording paradigm. Patch-clamp recordings were performed in the current-clamp mode either at the soma or at the dendrite (200 μm distance from the soma) proximal to the stimulating electrode. **b** Averaged sample traces recorded at the soma, showing EPSPs before and after Aβ_1–42_ peptide (500 nM) treatment. Potentiation of EPSPs was only detected when patch recording was performed at the soma, but not at the dendrite. **c**–**f** Graphs showing the time course and magnitude of the change in normalized EPSPs recorded at the soma (**c**, **d**) and at the dendrite where synaptic inputs were activated by stimulating electrode (**e**, **f**). **d**, **f** Summary of results from all experiments as that shown in (**c**) and (**e**), respectively. **g** Illustration of local application (pressure puffing) of GluN2B-NMDAR blocker ifenprodil (3 μM) to the soma. Whole-cell patch-clamp recording was performed at the soma while stimulation was delivered at the dendrite. **h** Aβ_1–42_-induced EPSP potentiation was reversed by the GluN2B-NMDAR blocker ifenprodil (3 μM) that was locally applied to the soma. Ifenprodil (3 μM) was applied 2 min after Aβ_1–42_ (500 nM) treatment. **i** Statistical histogram showing the dependency of the Aβ_1–42_-induced EPSP potentiation on the activity of somatic GluN2B-NMDARs. **j** Effects of Aβ_1–42_ at different concentrations on the potentiation of synaptic inputs. **k** Summarized dose–response data. **l** Similar potentiation effects produced by 100 nM TBOA and 500 nM Aβ_1–42_. Data are means ± SEM, **P < 0.01, *t* test; ns, no significance

As Aβ initiates the suppression of GLT as TBOA does, we examined whether Aβ can mimic TBOA’s effect by potentiating dendritic inputs at the soma (Fig. 6a). The acute hippocampal slices perfused with Aβ_1–42_ peptide (500 nM) yielded a progressive enhancement of evoked dendritic inputs (normalized potentiation magnitude, 1.77 ± 0.03, *n* = 7 from 7 rats, *P* < 0.01 Fig. 6b–d), indicating that Aβ_1–42_ treatment can potentiate dendritic inputs.

To further examine whether the Aβ_1–42_-mediated potentiation of dendritic inputs occurs at the dendrite where synaptic inputs were activated, we next performed patch-clamp recording at the dendrite proximal to the stimulating electrode (< 10 µm; Fig. 6a). The eEPSPs recorded at the stimulated dendrites 200 μm from the soma failed to exhibit any significant change during Aβ_1–42_ treatment (normalized potentiation magnitude, 0.98 ± 0.03, *n* = 4 from 4 rats, *P* < 0.01; Fig. 6b, e, f), suggesting that the modification of dendritic inputs occurred at the soma rather than the dendrite.

We further examined whether the Aβ_1–42_-mediated potentiation of dendritic inputs depended on the activation of NMDARs at the soma. As the TBOA-induced somatic potentiation of dendritic inputs depended on the activation of GluN2B-NMDARs at the soma, we performed a similar experiment by locally applying a GluN2B-NMDARs blocker ifenprodil (3 μM) to the soma of the recorded neurons 2 min after Aβ_1–42_ (500 nM) treatment (Fig. 6g). Similar to the previous findings, the Aβ_1–42_-induced somatic potentiation of dendritic inputs was totally abolished by ifenprodil treatment (normalized potentiation magnitude, 1.02 ± 0.02, *n* = 6 from 5 rats, *P* = 0.47; Fig. 6h, i). These results suggest that the Aβ_1–42_-induced potentiation of dendritic inputs depends on the activation of GluN2B-NMDARs located at the soma.

Aβ at 500 nM concentration induces somatic potentiation of synaptic inputs. Is this an all-or-none mechanism or is it dependent on the concentration of Aβ? To address this question, we performed a dose–response study in the key experiments and found that Aβ_1–42_ at 100 nM elicited potentiation of EPSPs with 1.29 ± 0.03 potentiation magnitudes (Fig. 6j, k). The Aβ_1–42_ peptide oligomer within the concentration range of 200–1000 nM (200, 500, 1000 nM) elicited potentiation of EPSPs with similar potentiation magnitudes (*P* > 0.05 among the concentrations, *t* test), indicating a stable potentiation magnitude at Aβ_1–42_ concentration of 200 nM and above. In contrast, Aβ_1–42_ at 50 nM failed to display any effect on EPSPs. Moreover, the magnitude of Aβ-induced potentiation was comparable to that induced by TBOA at 100 nM (Fig. 6l). These results indicate the incremental effect of Aβ_1–42_ is not an all-or-none mechanism but depends on the dosage of Aβ_1–42_.

Combined with the results in Fig. 5 showing the dependency of Aβ-mediated neuronal hyperactivity on elevated somatic NMDAR activation, these data suggest that the enhancement of sustained activation of NMDARs at the soma is the key mediator of the production of both somatic plasticity and hyperactivity. The somatic GluN2B-NMDAR-dependent potentiation mechanism is not only highly associated with but may also contribute to the Aβ-mediated neuronal hyperactivity.

## Discussion

By employing patch-clamp recordings on the soma or apical dendrite of the hippocampal CA1 pyramidal neurons, we revealed in the present study that the somatic potentiation of dendritic inputs may contribute to the neuronal hyperactivity caused by the impairment of glutamate reuptake. This somatic potentiation was accompanied by elevated tonic activity of NMDARs located at the soma. We further illustrated that the increased NMDAR activity at the soma  is essential to the somatic potentiation of dendritic inputs. Lastly, we examined these effects in the context of Aβ_1–42_ administration and found similar neural hyperactivity dependent on the NMDAR activity. This not only affirms the association between neural hyperactivity and amyloid plaques commonly observed in neurodegenerative diseases, but also suggests a novel pathological perspective that specifically involves the GluN2B-NMDAR-mediated potentiation at the soma.

### Deficit of GLT and neuronal hyperactivity in AD

Under normal circumstances, ambient extracellular glutamate is generally maintained at a relatively low level to ensure synaptic transmission with appropriate signal-to-noise ratio and to prevent excessive activation of GluRs. The appropriate amount of ambient glutamate is maintained by GLTs in glial cells *via* glutamate reuptake. Down-regulation of certain excitatory amino acid transporters (EAATs), such as GLT1, is often reported in various neuropsychiatric diseases such as epilepsy, stroke, AD, depression and movement disorders [3, 4]. Meanwhile, neuronal hyperactivity is also frequently observed in these neurological diseases [7, 42, 43]. Based on the excitatory nature of ambient glutamate, the causal link between GLT deficit and neuronal hyperactivity has been proposed [6]. As the down-regulation of EAATs causes accumulation of ambient glutamate at the extracellular milieu, which would likely in turn activate additional GluRs, we speculate that the changes in the membrane properties and the excitability of cells surrounded by the elevated ambient glutamate may contribute to the neuronal hyperactivity observed in various neuropsychiatric states.

In the present study, we determined the alterations of RMP, input resistance, spontaneous firing rate and threshold of AP generation in hippocampal CA1 pyramidal neurons following GLT blockade, and found progressive depolarization from RMP and increase in input resistance. Consistently, a substantial increase in the spontaneous spike firing and a dramatic decrease in the threshold of AP generation were also observed. Collectively, these results suggest that the changes in membrane and cell excitability are responsible for the neuronal hyperactivity elicited by down-regulation of GLT. Interestingly, all these changes induced by GLT blockade were totally reversed by pressure application of the NMDAR open channel blocker MK-801 onto the soma, pointing to a crucial role of somatic NMDAR activation in the generation of neuronal hyperactivity. We speculate that following chronic suppression of the GLTs, the accumulated ambient glutamate could diffuse from the synapse-rich dendrites to the soma to improve the tonic activation of somatic NMDARs.

### Tonic activation of somatic NMDARs and somatic potentiation of dendritic inputs

Currently, the widely accepted etiology for AD is that Aβ perturbs both synaptic transmission and plasticity [9, 10]. In the hippocampus of AD mice, for instance, enhanced neuronal activity has been detected [1, 2]. This neuronal overexcitation is related to the elevation of intracellular calcium concentration [44, 45]. On the other hand, Aβ oligomer has been reported to suppress the surface expression of NMDARs [46], hippocampal LTP, and learning and memory [47–49]. Notably, recent studies have further revealed that the Aβ-induced inhibition of LTP can be reversed by blocking extrasynaptic GluN2B-NMDARs, pointing to a more important role of extrasynaptic NMDARs in the production of abnormal plasticity [28, 50].

Here, we further demonstrated that the extrasynaptic NMDARs at the soma play a crucial role in both neuronal hyperactivity and correlated somatic potentiation. As Aβ suppresses GLT in glial cells, the accumulated ambient glutamate can lead to both tonic activation and desensitization of GluRs. We hypothesized that this effect may be mainly mediated by GluN2B-NMDARs. The GluN2B-NMDAR is not only more sensitive to glutamate compared to most other subtypes, but also preferentially located extrasynaptically at the soma [17, 22]. These somatic NMDARs are tonically activated by basal ambient glutamate [23, 24], an activity that has been shown to facilitate neuronal discharge at the soma and potentially contribute to the long-term hyperactivity [24, 30]. Consistent with our hypothesis, GLT suppression with TBOA or Aβ induced a substantial increment in NMDAR open probability at the soma and that such potentiation was totally reversed by somatic pressure applications of antagonists of GluN2B- (ifenprodil), but not of GluN2A-NMDARs. These findings provide evidence to support the correlation between somatic NMDAR activation and somatic potentiation of dendritic inputs, specifically unveiling the role of extrasynaptic GluN2B-NMDARs in neuronal hyperactivity. In addition, these results are consistent with our recent finding that dendritic inputs can undergo persistent modifications at the soma [30], which indicates a novel form of plasticity that also depends on the sustained activity of NMDAR at the soma. More importantly, in that study we have demonstrated that elevating the activation of somatic NMDARs by puffing NMDA to the soma is sufficient to induce somatic amplification of dendritic inputs. In this sense, the Aβ-induced somatic plasticity we revealed here could be a causative factor for neuronal hyperactivity in AD.

Based on the current results, we propose that the accumulated ambient glutamate caused by GLT blockade promotes the tonic NMDAR activity at the soma and in turn triggers a novel somatic amplification mechanism that accounts for the neuronal hyperactivity initiated by impaired glutamate reuptake in AD. The present findings drag our sight from synaptic to extrasynaptic compartment at the soma for the first time to illustrate the mechanisms underlying the Aβ-dependent neuronal hyperactivity. Future studies may seek the exact causal relationship between elevated glutamate tone, somatic NMDAR activation and neuronal hyperactivity by focal manipulations of glutamate levels or receptor functionality at the soma. Understandings of the somatic involvement in the overall electrophysiological activities of a neuron may shed light on how extracellular environment influences normal functionality and provide new therapeutic targets for amyloid plaque-induced pathologies.

## Conclusions

Our results reveal that suppression of glutamate uptake with either TBOA or human Aβ_1–42_ oligomer can elicit a novel form of neural plasticity that selectively occurs at the soma of CA1 hippocampal neurons. The somatic amplification of synaptic inputs may help elevate the cell excitability and thus contribute to the neuronal hyperactivity initiated by impaired glutamate reuptake in AD.

## Supplementary Information

**Additional file 1: Fig. S1**. Representative traces before and after various drug treatments. **Fig. S2**. SK channel is not implicated in TBOA-induced somatic amplification of synaptic inputs. **Fig. S3**. MK-801 at 10 μM exerts similar blocking effect as 50 μM MK-801 did on TBOA-induced potentiation in EPSPs. **Fig. S4**. L- and T-type calcium channels are not involved in TFB-TBOA-induced potentiation in EPSPs.

## Data Availability

The datasets used and/or analysed in this study are available from the corresponding author on reasonable request.

## Abbreviations

Aβ: β Amyloid
AD: Alzheimer’s disease
GluRs: Glutamate receptors
TBOA: TFB-threo-β-benzyloxyaspartic acid
NMDARs: N-methyl-D-aspartate receptors
GluN2B-NMDARs: GluN2B-containing NMDARs
GLTs: Glutamate transporters
EPSPs: Excitatory post synaptic potentials
