# Supplementary material for: NMDAR-dependent somatic potentiation of synaptic inputs is correlated with β amyloid-mediated neuronal hyperactivity
Source: Transl Neurodegener. 2021 Sep 8;10:34. doi: 10.1186/s40035-021-00260-3 (PMC8424869; doi:10.1186/s40035-021-00260-3)
Supplement: Supplementary file 1 — Additional file 1: Fig. S1. Representative traces before and after various drug treatments. Fig. S2. SK channel is not implicated in TBOA-induced somatic amplification of synaptic inputs. Fig. S3. MK-801 at 10 μM exerts similar blocking effect as 50 μM MK-801 did on TBOA-induced potentiation in EPSPs. Fig. S4. L- and T-type calcium channels are not involved in TFB-TBOA-induced potentiation in EPSPs. [file 40035_2021_260_MOESM1_ESM.docx]

**Supplemental figure and legends**


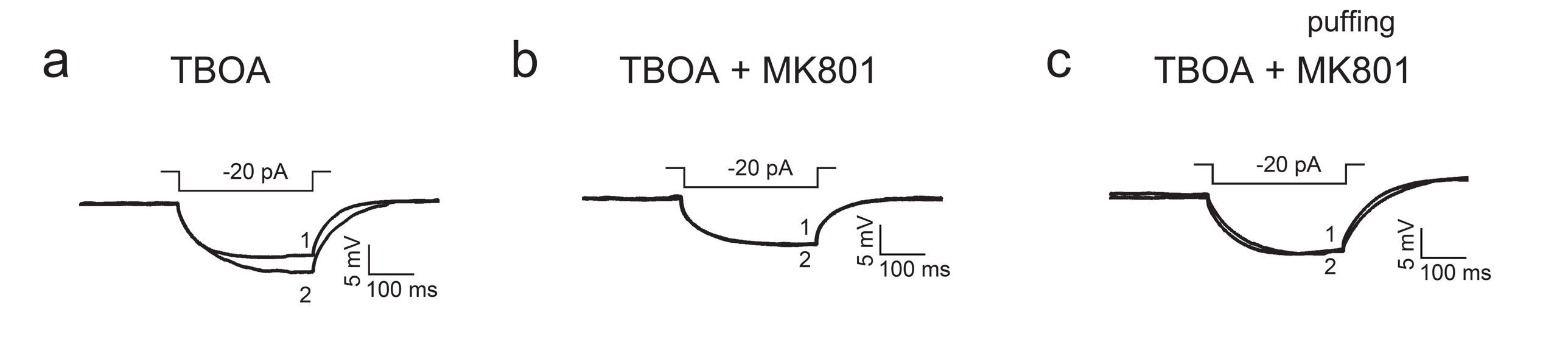


**Fig. S1**. Representative traces before (1) and after (2) various drug treatments showing increased input resistance following TBOA treatment (**a**) and reversal of this change following co-application of TBOA with MK-801 (50 µM) via perfusing slices (**b**) or via puffing the soma (**c**). Note that for these assessments, input resistance was measured by a single -20 pA current injection.


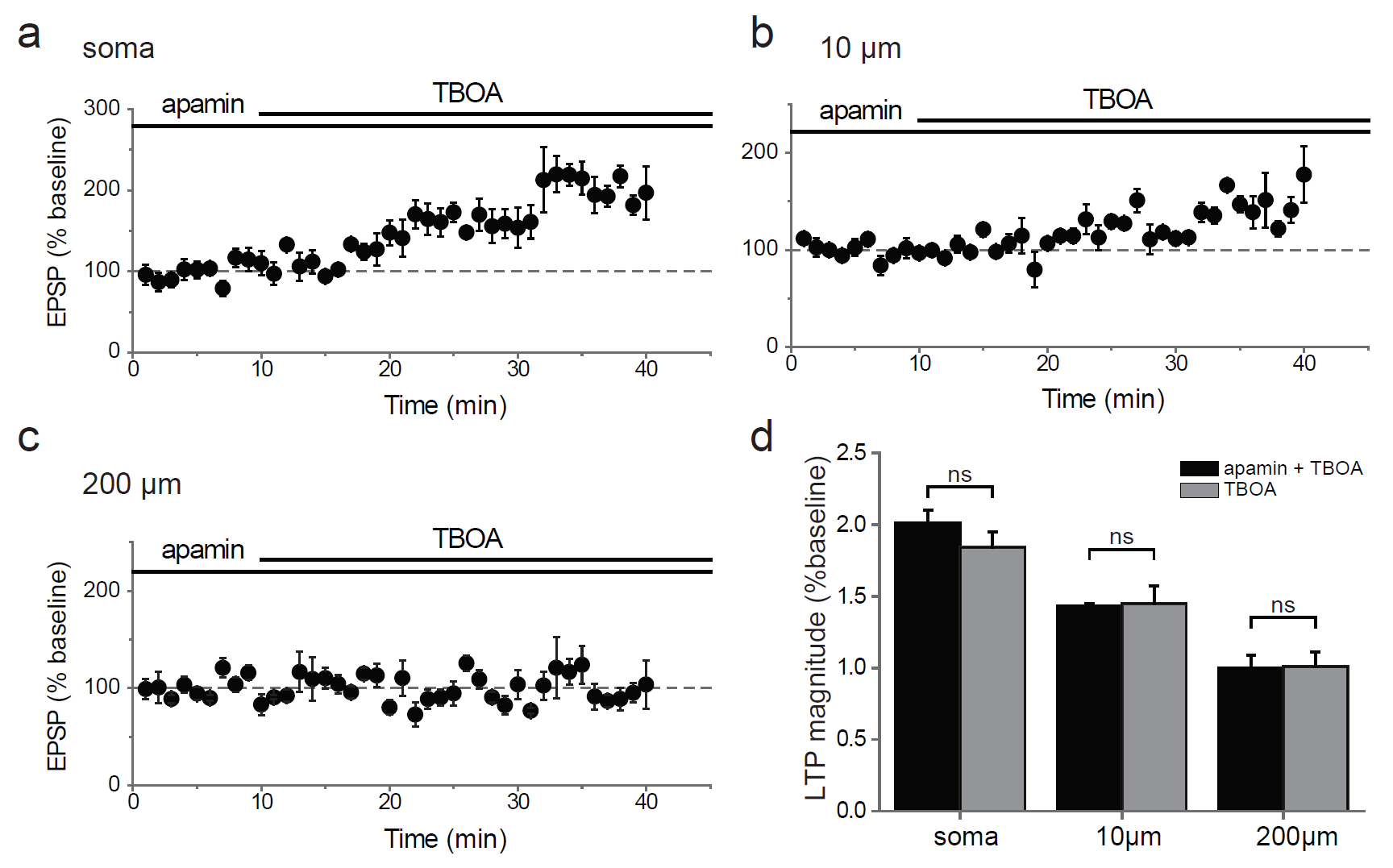


**Fig. S2**. SK channel is not implicated in TBOA-induced somatic amplification of synaptic inputs. (**a-c)**, A serial of patch recordings at the soma (a) or on dendrites located at 10 µm (b) or at 200 μm distance (c) from the soma reveals absence of effect by SK channel blocker apamin (100 nM) on TBOA-induced somatic amplification of dendritic inputs. (d) Histograms summarize the results in a-c. Soma, TBOA: 1.84 ± 0.11, *n =* 7 from 7 rats; apamin + TBOA: 2.01 ± 0.09, *n =* 5 from 4 rats, *P* > 0.05；dendrites (10 µm): TBOA: 1.45 ± 0.12, *n =* 4 from 4 rats; apamin + TBOA: 1.43 ± 0.02, *P* > 0.05, *n =* 4 from 4 rats; dendrites (200 µm): TBOA: 1.01 ± 0.08, *n =* 4 from 4 rats; apamin + TBOA: 1.01 ± 0.10, *n* = 4 from 4 rats; *P* > 0.05.


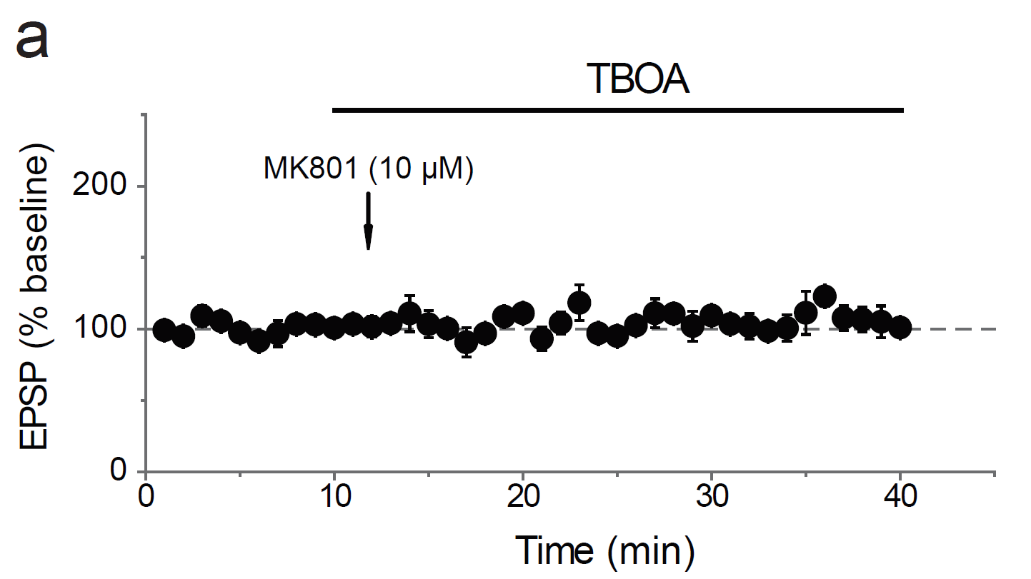


**Fig. S3**. MK-801 at 10 μM exerts similar blocking effect as 50 μM MK-801 did on TBOA-induced potentiation in EPSPs. TFB-TBOA-induced potentiation in EPSPs was reversed following locally applying of 10 μM MK-801 to block the activity of NMDARs located at the soma of recorded neurons.


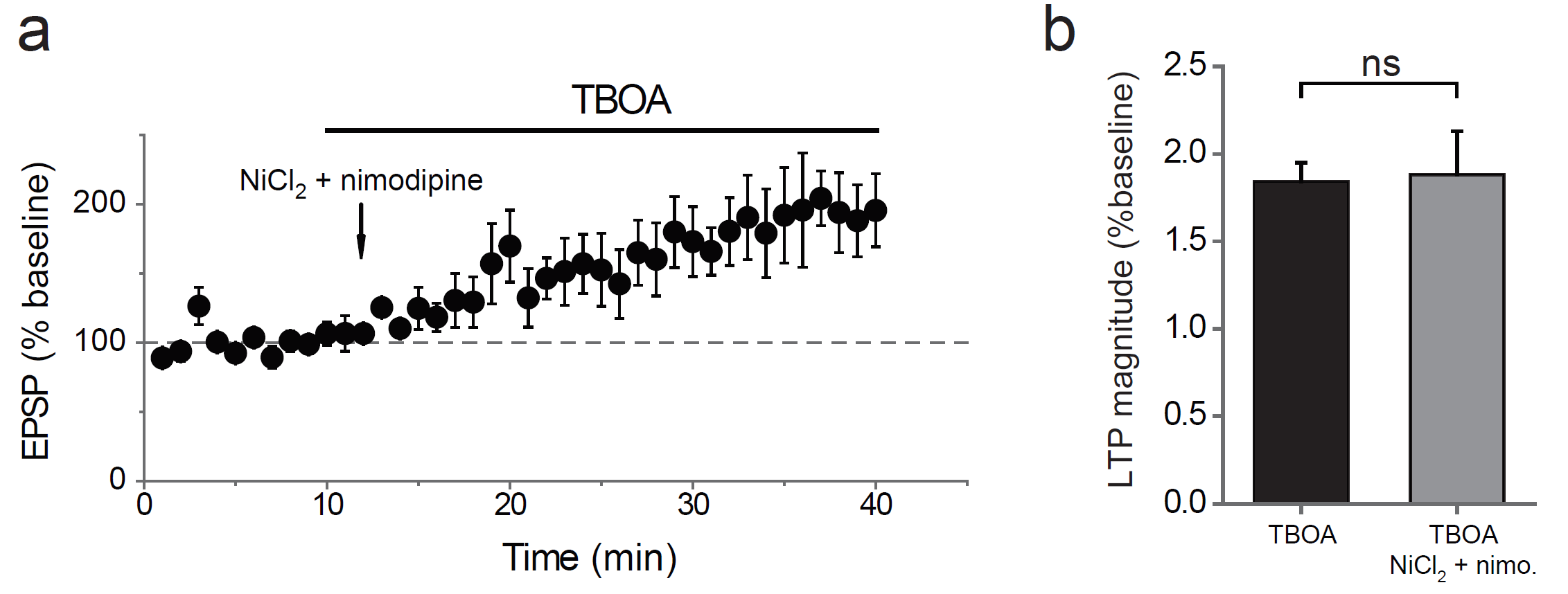


**Fig. S4**. L- and T-type calcium channels are not involved in TFB-TBOA-induced potentiation in EPSPs. (**a**) The change in EPSPs were monitored during which TFB-TBOA was applied with L-type calcium blocker nimodipine (10 μM) and T-type calcium blocker NiCl_2_ (100 μM). (**b**) No significant effects on the potentiation in EPSPs were observed following these treatments (NiCl_2_ + nimodipine, EPSP 1.88 ± 0.25, *n =* 6 from 4 rats, *P* > 0.05).
